# Incidence of scleritis requiring surgical repair from subconjunctival triamcinolone acetonide injections in patients with scleritis in TriNetX

**DOI:** 10.1186/s12886-026-05012-3

**Published:** 2026-06-10

**Authors:** Jesse Lama, Meghan Berkenstock

**Affiliations:** 1 Drexel University College of Medicine, Philadelphia, PA USA; 2 Division of Ocular Immunology, Wilmer Eye Institute, Johns Hopkins University School of Medicine, 600 N. Wolfe St., Maumenee 3rd Floor, Baltimore, MD 21287 USA

**Keywords:** Subconjunctival injection, Triamcinolone injection, Necrotizing scleritis, TriNetX, Scleral melt

## Abstract

**Purpose:**

To estimate the yearly and 15-year cumulative incidence of scleritis requiring surgical repair in patients with a prior diagnosis of scleritis who received a subconjunctival or subTenon triamcinolone acetonide injection (STAI).

**Design:**

A retrospective cohort study was conducted using the TriNetX US Collaborative Network. TriNetX is an electronic health records database with anonymized, deidentified encrypted data from 69 healthcare networks.

**Methods:**

Subjects with a history of scleritis were identified using ICD-10 (International Classification of Diseases, 10th Revision) code H15.0X and who subsequently underwent a STAI as identified using the CPT (current procedural terminology) code between January 1, 2009 to December 31, 2024. CPT codes were used to identify STAIs include subconjunctival injection (68200) or subTenon injection (67515) and injection of triamcinolone acetonide 10 mg (J3301). Please note that H15.0X codes specifying posterior scleritis were excluded from this cohort in an attempt to localize anterior scleritis. The time relation was set to ensure all patients in the cohort had an existing diagnosis of scleritis prior to any instance of STAI. The primary outcome measure was the annual and 15-year cumulative incidence of required surgical repair within 4 weeks, and 3, 6, 9, and 12 m after the STAI as best estimated by scleral graft reinforcement and repair of scleral staphyloma with graft, CPT 67255 or 66225. Final data collection ran on April 23, 2026. Possible confounding procedures such as cataracts were also excluded within the time period assessed in order to better localize the STAI for use in cases of scleritis.

**Results:**

Out of 113,510,724 patients on the TriNetX database, 36,249 had a diagnosis of scleritis. Of those with a history of scleritis, 176 had a subconjunctival or subTenon triamcinolone injection (STAI). Of this cohort, 0 patients needed surgical interventions requiring a patch graft over the 15-year time period of the study giving an annual and cumulative incidence of 0 per 100,000 persons. The subjects were primarily female (112, 63.64%) with a mean age of 61 years (range 14-90, SD 16).

**Conclusions:**

Surgical intervention requiring a patch graft was not observed post-injection among patients with prior scleritis who received a STAI. Further studies with larger cohorts are necessary to accurately represent the risk profile of STAI in the greater population.

## Introduction

Scleritis is an inflammatory condition of the sclera with an incidence of 3.4 to 7.6 per 100,000 in the United States [1]. In cases of treatment failure or contraindication of systemic immunosuppressants or corticosteroids, subconjunctival injections of triamcinolone acetonide have been used as an alternative therapy for non-necrotizing, focal, anterior scleritis. The development of necrotizing scleritis following a STAI has been documented in case reports or series [2–4]. The mechanism of scleral melt after triamcinolone injection has been posted as continued ischemia leading to scleral collagen matrix degradation given vasoconstrictive properties of triamcinolone [2, 3]. An additional concern is the development of infection with the STAI procedure, which again increases the risk of scleral melt.

While most reported cases of scleral necrosis after STAI occurred in patients without a prior history of scleritis, there is limited literature on the incidence of scleral melt after STAI with a pre-existing scleritis diagnosis. This study assessed the yearly and 15-year cumulative incidence of surgical intervention requiring a patch graft after STAI injection for the treatment of active scleritis in patients with and without a prior diagnosis of scleritis.

## Methods

The study was conducted through TriNetX, an electronic health records database with anonymized, deidentified encrypted data from 69 healthcare networks to comprise the TriNetX US Collaborative Network. ICD codes were used to identify subjects with scleritis (H15.0X), at any time prior to receiving a STAI from January 1, 2009 to December 31, 2024. Please note that H15.0X codes specifying posterior scleritis were excluded from this cohort in an attempt to localize anterior scleritis. CPT codes were used to identify STAIs: subconjunctival injection (68200) or subTenon injection (67515) and injection of triamcinolone acetonide 10 mg (J3301). Scleral melt following STAI administration was assessed through the need for the surgical placement of a scleral reinforcement graft/staphyloma graft: CPT 67,255 and 66,225 at 4 weeks, and 3, 6, 9, and 12 months post injection. Demographics collected included age, sex, race, and time from the diagnosis of scleritis to the administration of the STAI. Results with a value of less than ten in the TriNetX database are rounded to 10 in accordance with privacy statutes in the Health Insurance Portability and Accountability Act.

Categorical variables were reported as percentages and both the annual and cumulative incidence data was calculated using software on the TriNetX platform. This study was deemed exempt by the Drexel University College of Medicine Institutional Review Board and followed the Tenets of the Declaration of Helsinki.

## Results

**Fig. 1:**
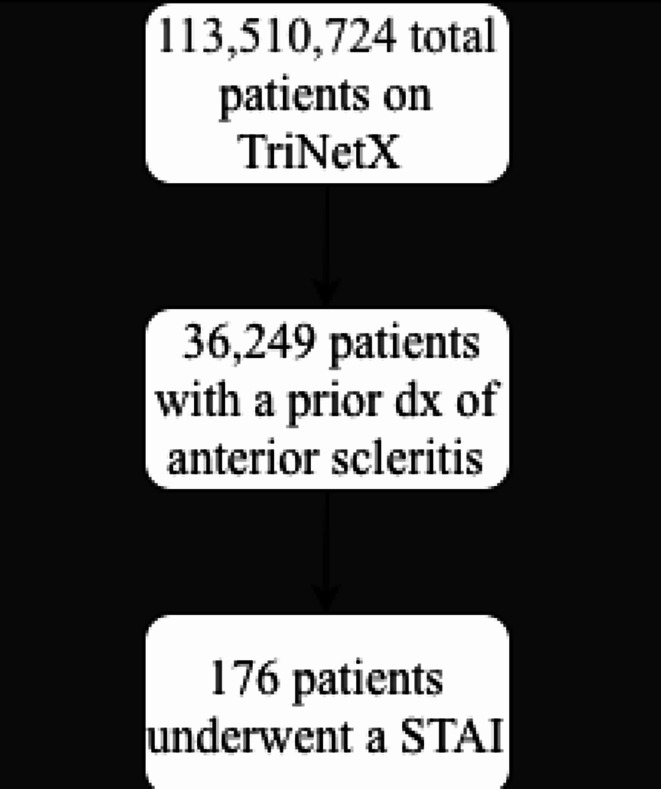
Flow chart representing patient cohort referenced

Out of 113,510,724 patients on the TriNetX database, 36,249 had a diagnosis of scleritis. Of those with a history of scleritis, 176 had a subconjunctival or subTenon triamcinolone injection (STAI). Of this cohort, 0 patients needed surgical interventions requiring a patch graft over the 15-year time period of the study giving an annual and cumulative incidence of 0 per 100,000 persons. The subjects were primarily female (112, 63.64%) with a mean age of 61 years (range 14–90, SD 16). (Fig. 1, line 140)

## Discussion

In the literature, there are few cases reported of necrotizing scleritis following a STAI injection [2–4]. Despite surgical removal of the triamcinolone deposit, scleral patch grafting may be required to preserve the structural integrity of the globe with transition to oral corticosteroids and non-corticosteroid immunosuppressants to manage the underlying scleritis. In a case study, a female patient was administered a STAI at the end of a vitreoretinal surgery and presented 1 month post-op with scleral melt and necrotizing scleritis. A scleral graft was placed and a week later, due to remaining epithelial damage, a triamcinolone acetonide deposit was removed [2]. It is evident here that use of scleral patch graft has been previously done after a STAI when complications of scleral melt arose, reaffirming our use of this as a surrogate code for scleral melt.

Still, the adverse event of scleral necrosis after STAI remains a rare complication as a retrospective study of 53 patients from 1999 to 2009 found the only adverse outcome was elevated intraocular pressure and no cases of scleral melt occurred [5]. Even with 29 months of follow-up, an additional study found that none of the 35 subjects who received a subconjunctival triamcinolone acetonide injection for non-necrotizing and non-infectious anterior scleritis developed scleral necrosis. The only documented adverse events included a subconjunctival haemorrhage, glaucoma or elevated intraocular pressures, or cataracts [6]. Another study positively quantified the improvement of non-necrotizing anterior scleritis after STAI in 97% of patient eyes with a mean follow up of 2.3 years. Furthermore, any recurrence of symptoms afterwards was reported as being correlated with the discontinuation of corticosteroids or immunosuppressants [7]. Our data mirror these findings with no cases of scleritis requiring a scleral patch graft placement post-STAI over 15 years.

While the database provides a nationwide cohort of over 120 million subjects from which to analyse the rare event of scleral melt, there are limitations to this study. A specific code for necrotizing scleritis is not available, which required use of a surrogate event, scleral graft placement, to assess for occurrence of the primary outcome. However, cases of necrosis could have occurred that were more mild and resolved without the need for graft placement. Furthermore, given the deidentified and aggregated nature of the data, analysis of individual medical records could not be performed to assess for the severity of the necrosis, the associated treatment methods, the time to resolution, or any associated pathologic studies and cultures. Small cohort rounding to 10 to protect patient privacy limited reporting of racial and ethnic demographics. Due to the nature of TriNetX coding and patient confidentiality, ocular laterality cannot be ensured within the study. Finally, the TriNetX database relies on ICD coding for data generation. Without retrospective chart analyses, coding errors cannot be excluded.

In summary, STAI administration for the treatment of non-necrotizing, non-infectious anterior scleritis resulted in no cases of necrotizing scleritis requiring scleral patch grafting. Given the rarity of scleral necrosis after an STAI, a large, multicentre retrospective study is needed to assess for the causes of scleral melt, treatments, and visual outcomes after STAI. While the development of scleral necrosis can occur, this study reinforces the exceptionally rare occurrence of this adverse event. Given the nature of the data collection and limitations of the TriNetX source which includes surgical repairs codes with a lack of laterality, causation, or more milder results, this study does not exclude the potential for milder complications. Likewise, the incidence reported does not account for all possible negative outcomes.

## Data Availability

The data that support the findings of the study are available from TriNetX platform but restrictions apply to the availability, as data were collected under license for the current study. The data, while not publically available, are available from authors upon reasonable request and permission of TriNetX.
